# Marine Bromophenol Derivatives as a Novel Class of Potent Small-Molecule STING Agonists

**DOI:** 10.3390/cimb48010061

**Published:** 2026-01-05

**Authors:** Manqing Tang, Qiuhui Guo, Ping Wang, Yunfei Li, Bo Jiang

**Affiliations:** 1 Laboratory of Experimental Marine Biology, Institute of Oceanology, Chinese Academy of Sciences, Qingdao 266000, China; 2 Nantong Zhong Ke Marine Science and Technology R&D Center, Nantong 226000, China; 3 College of Chinese Materia Medica, Tianjin University of Traditional Chinese Medicine, Tianjin 301617, China; tmq17836959048@163.com (M.T.); 15227606298@163.com (Q.G.); wangping81@tjutcm.edu.cn (P.W.)

**Keywords:** STING agonists, bromophenol derivatives, cancer immunotherapy

## Abstract

Activation of the stimulator of interferon genes (STING) pathway has emerged as a promising strategy for cancer immunotherapy. However, the initial cyclic dinucleotide (CDN) analogs developed as STING agonists have shown limited efficacy in clinical trials, prompting interest in non-CDN small-molecule alternatives. In this study, we identified a novel series of bromophenol derivatives as effective STING agonists. Among these derivatives, OSBP63 robustly activated the STING signaling pathway, resulting in enhanced phosphorylation of interferon regulatory factor 3 (p-IRF3) and increased secretion of interferon-β (IFN-β). Co-administration of Marine Bromophenol Derivative (OSBP63) with paclitaxel (PTX), a conventional anticancer drug, significantly suppressed B-cell lymphoma-2 (BCL-2) expression and protein kinase B (AKT) phosphorylation, thereby demonstrating pronounced anti-tumor activity in a mouse model of breast cancer. These findings suggest that OSBP63 represents a promising non-CDN small-molecule STING agonist candidate, offering a valuable lead for future anticancer therapeutic development.

## 1. Introduction

The stimulator of interferon genes (STING) is a central adaptor that governs innate immune detection of cytoplasmic DNA and initiates robust anti-tumor and anti-pathogen responses. When cyclic GAP-AMP synthase (cGAS) senses aberrant cytosolic double-stranded DNA (including tumor-derived DNA), it generates the second messenger cyclic GMP-AMP (cGAMP), which binds to STING on the endoplasmic reticulum membrane [1,2]. Ligand engagement triggers STING activation translocation to perinuclear compartments, where it recruits and activates TANK-binding-kinase 1 (TBK1). TBK1 subsequently phosphorylates interferon regulatory factor 3 (IRF3), enabling IRF3 dimerization and nuclear translocation to induce type I interferons such as interferon-β (IFN-β). In parallel, STING signaling activates the nuclear factor-κB (NF-κB) pathway, amplifying pro-inflammatory cytokine production [3,4,5]. Together, the cGAS–STING axis coordinates the innate immune program that underpins subsequent adaptive immunity.

Given its pivotal role in orchestrating innate and adaptive immunity, particularly against infections and cancer, STING has become an attractive therapeutic target [6,7]. Although endogenous cyclic dinucleotides (CDNs) such as cGAMP activate STING, their limited membrane permeability and rapid degradation constrain clinical efficacy and have yielded modest response rates [8]. To overcome these limitations, Fu et al. designed the first generation CDN analogs by modifying the phosphodiester backbone of cGAMP, including ADU-S100 and Ulevostinag (MK-1454) [9,10,11]. This modification enhanced stability and cell membrane permeability, thereby increasing the analogue’s ability to induce IFN-β secretion. Both compounds share the intrinsic limitations of the CDN scaffold, including suboptimal cellular uptake and metabolic stability [12]. TAK-676, a systemically deliverable CDN-like agonist, has demonstrated durable antitumor responses and robust type I interferon induction in multiple syngeneic models and is now in phase I trials [13]. These examples highlight both the progress and the persistent formulation and delivery challenges that must be addressed to fully realize STING-targeted therapeutics.

The development of non-CDN STING agonists has become a focal point of antitumor drug research, particularly since GlaxoSmithKline reported diABZI—an amidobenzimidazole (ABZI) dimer—in 2018. In diABZI, two symmetry-related ABZI pharmacophores are joined by a four-carbon linker that mimics the native STING dimer interface, thereby markedly enhancing binding affinity, cellular uptake, and pharmacokinetic properties [14]. This innovation addressed several limitations of CDN-based agonists such as ADU-S100, whose clinical progress has been constrained by poor physicochemical stability and suboptimal efficacy. Subsequent ABZI analogs with potencies surpassing that of cGAMP have consolidated non-CDN agonists as a promising new class of synthetic STING activators. The discovery of diABZI also stimulated broader efforts that yielded compounds such as MSA-2, an orally bioavailable small molecule that engages STING as a non-covalent dimer and elicits robust antitumor immunity in murine colorectal cancer models [15]. Nevertheless, interim phase I data indicate that systemic non-CDN STING agonists still deliver only modest clinical benefit, highlighting the need for additional low-molecular-weight scaffolds with improved efficacy and safety profiles. Progress is further hampered by the scarcity of publicly available structure–activity relationship studies on dimeric STING agonists [16].

Bromophenols, abundant secondary metabolites from Rhodophyta and, to a lesser extent, brown and green macroalgae, possess diverse bioactivities—including antioxidant, anti-inflammatory, antimicrobial, and anti-diabetic effects [17]. Importantly, several bromophenols exhibit multitarget antitumor properties by modulating kinase activity, inducing apoptosis, and reprogramming cellular metabolism [18,19]. However, their capacity to engage the STING pathway has not yet been investigated. The present study therefore explores marine-derived bromophenols as a novel class of non-CDN STING agonists, aiming to expand the chemical space of STING modulators and overcome the limitations of current ABZI-based compounds.

## 2. Materials and Methods

### 2.1. Chemistry

All of the bromophenol compounds were prepared according to the literature Procedures [20]. The following chemical reagents were all purchased from TCI (Shanghai) Development Co., Ltd. (Shanghai, China) 1,2-dimethoxybenzene was acylated with 3,4-dimethoxybenzoic acid in the presence of polyphosphoric acid to afford bis-(3,4-dimethoxy-phenyl)-methanone, which was regarded as the crucial intermediate for synthesize varying position and number of bromine substitution on the phenyl ring. Reductions of diarylketone compounds were carried out with triethylsilane in trifluoroacetic acid to obtain the corresponding diarylmethane compounds. Finally, demethylation of both diarylketone and diarylmethane compounds with boron bromide in dichloromethane cleanly gave corresponding bromophenols compounds in good yields (The synthetic route is shown in Figure S1 of the Supplementary Information).

### 2.2. Materials and Animals

The 4T1 and E0771 cells were purchased from Ubigene Biosciences Co., Ltd. (Guangzhou, China). THP1 were obtained from the Cell Resource Center, Peking Union Medical College (Beijing, China). Fetal bovine serum (FBS) was provided by Pusaier Life Technology Co., Ltd. (Wuhan, China). Matrigel (catalog number: 082724) was purchased from Shanghai Nova Medical Technology Co., Ltd. (Shanghai, China). Roswell Park Memorial Institute (RPMI) 1640 was purchased from Gibco Thermo Fisher Scientific Co., Ltd. (Shanghai, China). The diABZI was purchased from MedChemExpress (Shanghai, China). The bicinchoninic acid (BCA) kit were purchased from Tiangen Biotech Co., Ltd. (Beijing, China). The STING, p-STING, p-STING, p-IRF3, BCL-2, p-AKT and β-actin antibodies were purchased from Cell Signaling Technology, Inc. (Shanghai, China). The agarose and low-melting-point agarose were purchased from Yien Chemical Technology Co., Ltd. (Shanghai, China). Paclitaxel (PTX) was purchased from Vokai Biotechnology Co., Ltd. (Beijing, China).

Female *BALB/c* and *C57BL/6J* mice, aged 6–8 weeks at the start of each experiment, were purchased from Beijing Vital River Laboratory Animal Technology Co., Ltd. (Beijing, China).

### 2.3. Molecular Docking

The marine bromophenols were docked to the crystal structures of STING proteins in both their closed and open forms, using 2′,3′-cGAMP and diABZI as positive controls. Protein Preparation: The crystal structures of the STING proteins (4LDH and 6DXL) were obtained from the RCSB PDB website. Water molecules and ligands were removed using Discovery Studio 2019 software, which also facilitated pre-treatments like hydrogenation to optimize the receptor state. Small Molecule Preparation: The chemical structures of the marine bromophenols were created using ChemDraw 20.0. Energy minimization was performed with Chem3D 20.0. software to achieve an energy-optimal steady state, after which the structures were imported into Discovery Studio 2019 for further pretreatment. Docking scores were calculated using CDOCKER in Discovery Studio 2019. Conformations with optimal binding energies were chosen for analysis and visualization with PyMOL 2.6.0. software.

### 2.4. Western Blot Assay

Protein was extracted from 4T1/THP-1 cells treated with either diABZI or marine bromophenols. After quantifying the protein concentration using the BCA kit, the proteins were separated by 10% sodium dodecyl sulfate-polyacrylamide gel electrophoresis (SDS-PAGE) and then transferred to polyvinylidene fluoride (PVDF) membranes (Millipore, Burlington, MA, USA). The membranes were blocked with PBS containing 5% skim milk powder and incubated overnight with the following primary antibodies: STING, p-STING, p-TBK1/NAK, p-IRF3, p-AKT, BCL-2 and β-actin, all at a dilution of 1:1000. Following this, the membranes were washed and incubated with an enzyme-labeled secondary antibody for 1 h. Finally, protein bands were visualized using a BioRad ChemiDoc MP imaging system (Bo Le Life Medical Products Co., Ltd., Shanghai, China).

### 2.5. Soft Agar Colony Formation Assay

Preparation of Lower Agarose Gel: Prepare an agarose solution using deionized water at a concentration of 1.2%. After autoclaving, add an equal volume of RPMI-1640 medium containing 20% FBS, mix thoroughly, and add 3 mL to each Petri dish. Allow the mixture to solidify to form the lower gel layer. Preparation of Upper Agarose Gel: Prepare a low-melting-point agarose solution at 0.8% concentration using the same deionized water. After autoclaving, add an equal volume of RPMI-1640 complete medium with 20% FBS and cells (2.5 × 10^3^ cells per dish) and mix thoroughly. Then, add 3 mL of this mixture to the solidified lower gel. The gel should be incubated at 4 °C until completely solidified and then covered with 3 mL of complete medium containing 10% FBS. Different concentrations of marine bromophenols were added as intervention drugs based on the experimental groups. The medium was changed weekly to maintain cell viability. After four weeks of continuous incubation, discard the upper layer of the medium. Fix the gel with methanol, stain with crystal violet, and collect images of the samples. Finally, count the number of cell colonies using Photoshop 2020 image analysis software.

### 2.6. RT-qPCR Assay

Marine bromophenols-treated 4T1 cells were collected, and total RNA was extracted using Trizol reagent (Invitrogen, Waltham, MA, USA). The RNA concentration was quantified using a NanoDropTM One (Thermo Fisher Scientific Inc., Waltham, MA, USA). cDNA was synthesized for qPCR amplification using an RT mix with DNase (All-in-one) (Beijing Bozhiyuan Biotechnology Co., Ltd., Beijing, China). Subsequent amplification was conducted on an ABI QuantStudio 3 system using the ABI TaqMan Gene Expression Assay to analyze *GAPDH* levels and standardize mRNA expression levels of specific genes. The expression levels of *CCL4*, *CXCL9*, and *CXCL10* were quantified using SYBRgreen qPCR Mix (MedChemExpress, Shanghai, China) and a 96-well real-time fluorescence quantitative PCR instrument (Thermo Fisher Scientific Inc., USA). The primer sequences utilized were as follows: 5′-TTCCTGCTGTTTCTCTTACACCT-3′ and 5′-CTGTCTGCCTCTTTTGGTCAG-3′ (*CCL4*), 5′-AACGTTGTCCACCTCCCTTC-3′ and 5′-CACAGGCTTTGGCTAGTCGT-3′ (*CXCL9*), 5′-GCCGTCATTTTCTGCCTCA-3′ and 5′-CGTCCTTGCGAGAGGGATC-3′ (*CXCL10*), 5′-GGTGAAGGTCGGTGTGAACG-3′ and 5′-CTCGCTCCTGGAAGATGGTG-3′ (*GAPDH*).

### 2.7. Enzyme Linked Immunosorbent Assay

The quantification of IFN-β in cell supernatants was performed using the Quantikine^®^ Human IFN-β Immunoassay Kit (Sangon Biotech Co., Ltd., Shanghai, China) as follows: First, the cell culture medium treated with the marine bromophenols was centrifuged, and the supernatant was collected as the sample solution. Next, 50 μL of Assay Diluent was added to the reaction wells, followed by the addition of blank diluent, standard, and sample solutions to the corresponding wells. The wells were then sealed with sealing paper and incubated at room temperature for 2 h. After washing the plate, 200 μL of IFN-β antibody solution was added to the reaction wells, which were then sealed and incubated at room temperature for 2 h. The plate washing procedure was repeated, after which the prepared reaction substrate was added to the reaction wells and incubated for 30 min at room temperature, protected from light. Following the reaction, 50 μL of termination solution was added. After gentle mixing, the absorbance at 450 nm (A450) was immediately measured using a multifunctional microplate reader (Synergy H1, Bio Tek Instruments, Inc., Winooski, VT, USA).

### 2.8. Cellular Thermal Shift Assay (CETSA)

The target binding properties of the marine bromophenols to STING proteins were assessed using a cellular thermal shift assay. The DMSO (Concord Technology Co., Ltd., Tianjin, China) group served as the control, and the marine bromophenols-treated cells were collected. The cell precipitates were then resuspended in PBS containing a Protease Inhibitor Mix (PIR) (Proteintech Group, Inc., Wuhan, China). The cell suspension was dispensed into eight centrifuge tubes, each containing 100 mL (approximately 1 × 10^6^ cells). A specific temperature was assigned to each tube and heated precisely in a metal bath for 3 min. The centrifuge tubes were then subjected to two cycles of freezing and thawing in liquid nitrogen. After the cell lysates were collected, the samples were centrifuged for 20 min at 4 °C and 12,000 rpm to remove cellular debris. Subsequently, a sampling buffer was added to each tube, and the samples were heated continuously in a water bath at 100 °C for 7 min to fully denature and inactivate the proteins. The resulting samples were then processed using the same procedure as Western blotting to obtain the protein band images. The expression intensity of the target proteins was analyzed using ImageJ 2.12.

### 2.9. Cell Proliferation Inhibition Assay

The MTT assay was used to analyze the proliferation inhibitory properties of the marine bromophenols on breast cancer cells. Cells in logarithmic growth phase were inoculated in 96-well plates at a certain density. The inoculated cells were incubated in 5% CO_2_, 37 °C incubator overnight. Then, different marine bromophenols suspensions were added to the culture wells and the cells were incubated in the incubator. At the end of incubation, 50 µL of 5 mg/mL MTT (Merck & Co., Inc., Rahway, NJ, USA) solution was added to each well, and then after 4 h of incubation at 37 °C, 200 µL of DMSO was added to each well, and then incubated at 37 °C for another 1 h to fully dissolve the Formazan crystals. The absorption value (A) of each culture well was determined at 570 nm using an enzyme labeling apparatus.

### 2.10. Investigating the Effects of OSBP63 on a Mouse Model of Transplantable Breast Cancer

We established a breast cancer homograft tumor model to evaluate the therapeutic effect of OSBP63 in a mouse model of breast cancer (E0771). A total of 5 × 10^5^ E0771 cells were injected subcutaneously into the axillary mammary fat pads of the *C57BL/6J* mice. OSBP63 was administered via intra-tumor injection at a dose of 5 mg/kg when the tumors reached approximately 100 mm^3^. Tumor growth was monitored on a weekly basis. Tumor size was measured using digital vernier calipers. When the tumor volume reached approximately 2500 mm^3^, the mice were euthanized by cervical dislocation, and the tumor tissue was subsequently excised and photographed for documentation.

### 2.11. Pharmacodynamic Evaluation of OSBP63 Combined with PTX in an In Vivo Breast Cancer Model

An orthotopic breast cancer model was developed by injecting 5 × 10^5^ 4T1 cells subcutaneously into the fourth mammary fat pad of BALB/c mice. When the tumors reached approximately 100 mm^3^, they were randomly assigned to three groups: a Control group, a PTX group, and an OSBP63 + PTX group. OSBP63 and PTX were administered intravenously at doses of 5 mg/kg and 2 mg/kg, respectively, while saline served as the control. Tumor growth was measured weekly using vernier calipers. Mice were euthanized when the tumor volume reached approximately 2500 mm^3^, and tumor tissues were subsequently excised and photographed.

### 2.12. Statistical Analysis

All statistical analyses were performed using GraphPad Prism version 9.5.0 software. One-way ANOVA combined with the Tukey post hoc test was used for comparisons among multiple groups. Statistical significance was defined as *p* < 0.05.

## 3. Results and Discussion

### 3.1. Investigation of Bromophenol Derivatives as High-Affinity Ligands for the STING Protein

**Figure 1:**
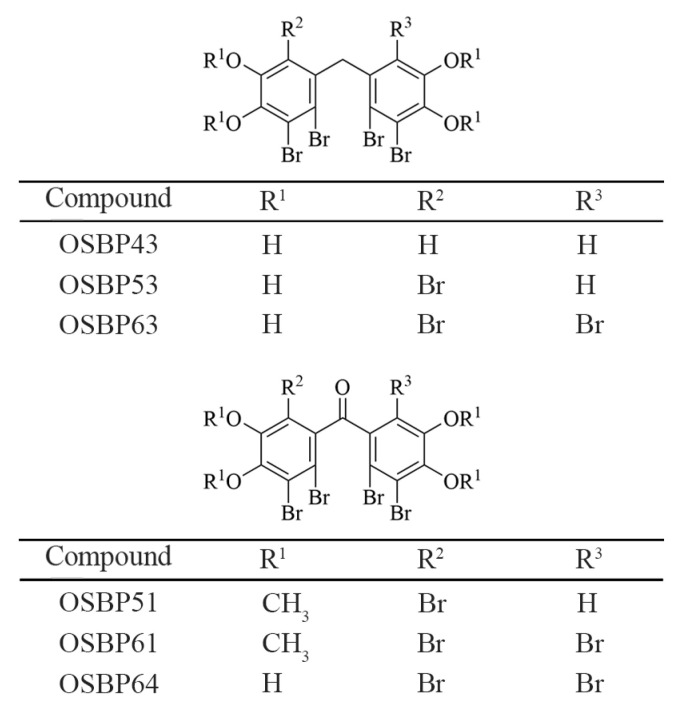
Chemical structures of the marine bromophenol OSBP43 and its derivatives.

Building on the structures of the non-CDN STING agonists diABZI and MSA-2, we hypothesize that symmetric compounds bind the butterfly-shaped STING protein more efficiently. We propose that ligands whose key moieties lie four to six atoms from the STING dimer interface display maximal activity. Notably, OSBP43—a natural bromophenol from marine red alga—matches this structural criterion. To test this hypothesis and explore broader structure-activity trends, we synthesized a panel of bromophenol derivatives (Figure 1).

**Figure 2:**
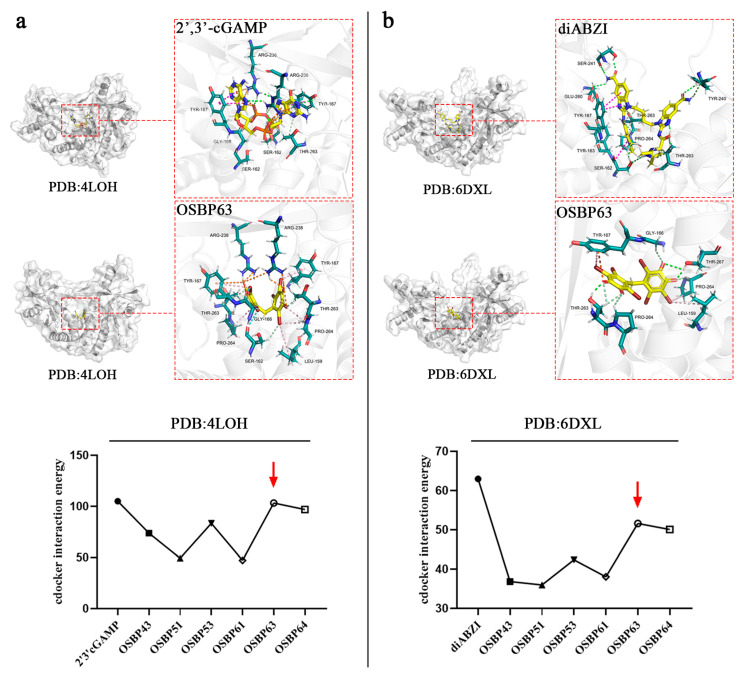
Marine bromophenols were docked to STING with Discovery Studio, and the results were visualized in PyMOL. (**a**) The absolute value of the binding energies for bromophenols docking to STING–cGAMP (PDB:4LOH). (**b**) The absolute value of the binding energies of bromophenols docking to STING–diABZI (PDB:6DXL). (Since the binding energy is negative (a more negative value indicates stronger binding), the results are expressed using the absolute value of the binding energy).

2′,3′ cGAMP, a natural STING agonist, induces a conformational shift in the STING dimer that activates downstream signaling. Recent crystal structures show that the STING dimer can adopt active closed and open conformations, as exemplified by the agonist diABZI. We docked each bromophenol derivative into closed and open STING structures (PDB: 4LDH and 6DXL) with 2′,3′ cGAMP and diABZI as positive controls. The results show that OSBP63 exhibits strong binding affinity to both STING conformations (Figure 2). Specifically, the stable binding of OSBP63 to STING relies on multiple intermolecular interactions, mainly including (i) hydrogen bonds formed between the molecule’s hydroxyl groups and STING residues THR263 and THR267 and (ii) the bromine atom forming halogen bonds with residues GLY166 and SER162 and also engaging TYR167 via π-stacking. The synergistic effect of these intermolecular forces significantly enhances the stability of the complex, demonstrating that OSBP63 has an excellent binding affinity for STING. OSBP43 displayed lower affinity, attributable to missing bromine atoms at the R2 and R3 positions. OSBP51 and OSBP61 bound even more weakly; methoxy substitution of the phenolic hydroxyl group abolished key hydrogen bonds. These results underscore the superior binding of the bromophenols to STING dimers. An MTT assay demonstrated that OSBP53 and OSBP63 induced apoptosis in 4T1 and E0771 cells at 0.5 μM (Figure S2 in Supplementary Information). These findings highlight the bromophenols as promising non-nucleotide STING agonists worthy of further therapeutic development.

### 3.2. Investigation of Bromophenol Derivatives Targeting the cGAS–STING Pathway

**Figure 3:**
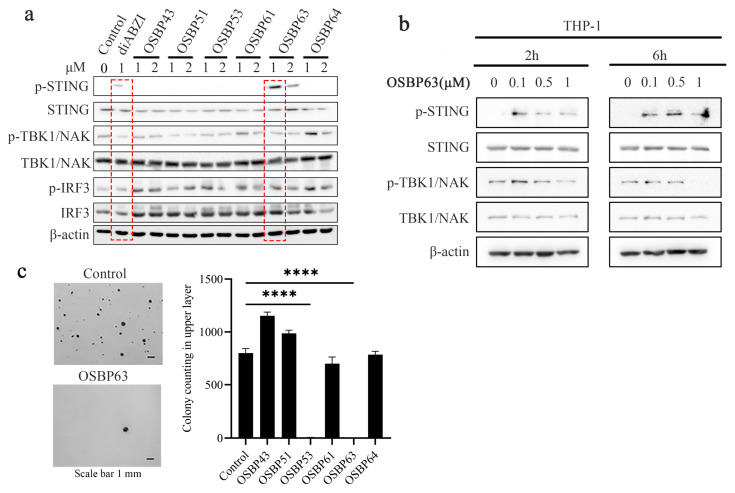
(**a**) 4T1 cells were treated with marine bromophenols (1 µM or 2 µM) for 24 h. Protein levels of phosphorylated STING (p-STING), TBK1 (p-TBK1), and IRF3 (p-IRF3) were analyzed by Western blotting (The band quantitative analysis graph is presented in Supplementary Figure S5). (**b**) THP-1 cells were treated with OSBP63 (0.1 µM, 0.5 µM or 1 μM) for 24 h. Protein levels of phosphorylated p-STING, p-TBK1, and β-actin were analyzed by Western blotting (The band quantitative analysis graph is presented in Supplementary Figure S5). (**c**) 4T1 cells were exposed to marine bromophenols (2 µM) and evaluated using a soft agar colony formation assay. Colonies formed were quantified, and results are shown as bar graphs (*n* = 3; **** *p* < 0.0001 vs. control).

To clarify the mechanism of bromophenols activating the cGAS–STING pathway, we carried out Western blotting of key signaling proteins. 4T1 cells were exposed to graded concentrations of the bromophenols for 24 h, and levels of p-STING, p-TBK1, and p-IRF3 were quantified. The diABZI was used as the positive control. As shown in Figure 3a, OSBP63 uniquely enhances STING phosphorylation and upregulates p-IRF3 expression. In contrast, the other marine bromophenols did not noticeably elevate p-STING levels. IRF3 acts downstream of STING, and its phosphorylation serves as a key readout of pathway activation, further supporting OSBP63’s ability to activate STING.

To further validate the activation of the STING pathway by this compound, we replicated the experiment in human THP-1 monocytes. The THP-1 cell line naturally harbors a functional h-STING gene, enabling endogenous h-STING protein expression with native structure and signaling [21]. Under standard conditions, h-STING in THP-1 cells stays at basal levels without spontaneous overactivation. Activation via phosphorylation only occurs upon specific stimuli, like extracellular cGAMP transfection, DNA virus infection, or STING agonist treatment [22]. In this study, THP-1 cells were treated with OSBP63 at 0.1, 1, and 5 μM under identical conditions. Results showed that OSBP63 significantly increased p-STING and p-TBK1 levels at 0.1 μM (Figure 3b), confirming activation of the human STING pathway. However, in murine cells, STING activity declines significantly as OSBP63 concentration increases. This concentration-dependent inhibition has also been reported for the clinical STING agonist ADU-S100 [23]. We speculate that, at high concentrations, the compound may weaken STING pathway activation by non-specifically activating certain kinases. It remains unclear which specific molecular mechanisms and kinases regulate the phosphorylation of high-concentration OSBP63. Furthermore, under high concentrations, the compound affects AKT phosphorylation (see Figure a in Section 3.6). Based on this, we further speculate that the compound may also weaken STING activation through other, as yet unclarified, molecular mechanisms.

Defective cGAS–STING signaling promotes tumorigenesis [24]. Beyond immune modulation, cGAS–STING directly triggers senescence and apoptotic programs in cancer cells [25,26]. Pathway activation increases pro-apoptotic BH3 proteins in a p53-independent manner and concurrently down-regulates BCL-2 while up-regulating BAX [27]. BAX activation permeabilizes the mitochondrial outer membrane, enabling caspase-9–mediated caspase-3 activation and culminating in apoptosis [28]. Thus, an intact cGAS–STING axis is pivotal for controlling cancer cell growth, senescence, and immune surveillance. We subsequently assessed the impact of STING activation on the tumorigenic potential of 4T1 cells using a three-dimensional tumor-cell culture model. The results from Supplementary Figure S3 (soft agar colony formastion assay) and Supplementary Figure S4 (sphere formation assay) were highly consistent, confirming the inhibitory effect of STING activation on tumor cell growth. Specifically, as shown in Figure 3c, OSBP53 and OSBP63 significantly reduced 4T1 colony formation relative to other bromophenols, indicating potent tumor growth inhibition.

### 3.3. Mechanistic Study on STING Activation Induced by Bromophenol Derivatives

Cancer often arises due to suppressed immune responses, which hinder the ability of effector cells such as tumor-specific CD8^+^ T cells to resist tumor proliferation [29]. Cancer immunotherapy seeks to revive and sustain the host immune system for the targeting and eradication of tumor cells. Research highlights the significance of the STING pathway in innate immune recognition of cancer. Studies demonstrate that mice deficient in TLR or MyD88 exhibit typical CD8^+^ T cell responses, while those lacking STING and IRF3 show impaired initiation of CD8^+^ T cells and fail to reject immunogenic tumors [29]. STING activation triggers IRF3 nuclear translocation, leading to the production of type I interferons like IFN-β. Fuertes’ team [30] identified host type I interferons as crucial mediators in the spontaneous onset of anti-tumor CD8^+^ T cell responses, bridging the gap between tumor recognition and adaptive T cell responses. Therefore, activating the STING pathway is crucial for initiating a potent tumor-specific T cell response that can directly eliminate tumor cells [31].

**Figure 4:**
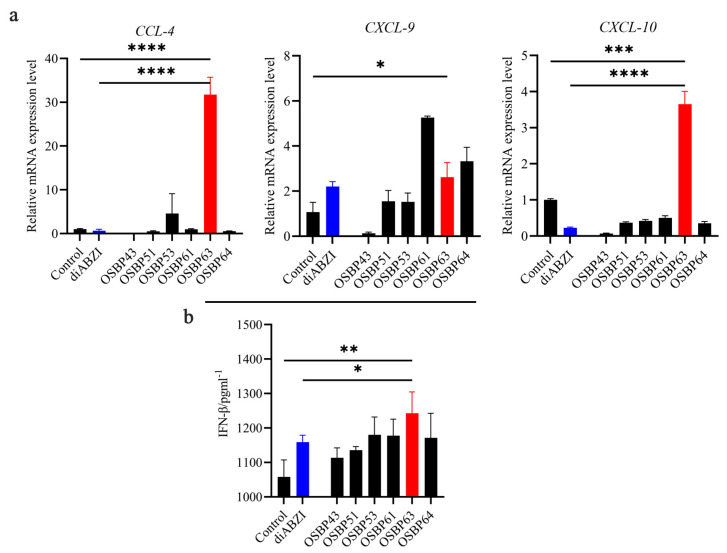
(**a**) 4T1 cells were treated with marine bromophenol (1 µM, 24 h), and mRNA levels of target genes were quantified (*n* = 3; * *p* < 0.05, *** *p* < 0.001, **** *p* < 0.0001 vs. control; **** *p* < 0.0001 vs. diABZI). (**b**) 4T1 cells exposed to marine bromophenol (1 µM, 24 h) were analyzed for IFN β secretion following STING activation (*n* = 3; ** *p* < 0.01 vs. control; * *p* < 0.05 vs. diABZI).

Chemokines constitute a family of cytokines that elicit chemotaxis in cells. Following receptor binding, they participate in diverse physiological processes, including immune responses, tumor proliferation, differentiation induction, and metastasis. Among human chemokines, a co-regulated group of three (*CCL4*, *CXCL9*, and *CXCL10*) shows a significant correlation with CD8^+^ T cell infiltration. Furthermore, this signature of three chemokines distinguishes tumors with increased T cell activation scores, including MHC I presentation and T cell/APC costimulation, as well as enhanced expression of innate immune sensing pathways implicated in T cell priming, such as the STING inflammasome pathway [32]. Importantly, type 1-IFN upregulates the expression of *CCL4*, which is crucial for recruiting inflammatory monocytes to the tumor site [33]. In our study, OSBP63 significantly increased the mRNA expressions of *CCL4* (>30-fold), *CXCL9* (>1.1-fold), and *CXCL10* (>30-fold) compared to the positive control diABZI, a known STING agonist, with notable effects observed for *CCL4* and *CXCL10* (Figure 4a). However, diABZI failed to significantly up-regulate the expression of *CCL4* and *CXCL10*, a phenomenon closely related to the branching regulatory mechanism of the STING pathway. After STING activation, TRAF6 must be recruited with IRF3 as the adaptor protein to initiate the NF-κB pathway and mediate the expression of chemokines *CCL4* and *CXCL10*. This process exhibits a clear spatiotemporal distinction from the type I interferon (such as IFN-β) response mediated by IRF3 dimerization. Therefore, it can be inferred that diABZI-induced STING conformational changes preferentially trigger IRF3 dimerization signaling at the Golgi apparatus rather than the NF-κB activation process located at endolysosomes [34], ultimately leading to a weak induction of *CCL4* and *CXCL10*.

Current research generally holds that adequate expression of *CCL4* and *CXCL10* in tumor tissues is a prerequisite for overcoming the immunosuppressive “cold tumor” microenvironment and achieving efficient CD8^+^ T cell infiltration [35]. Insufficient chemokine induction is a key bottleneck in the clinical translation of some STING agonists [36]. To definitively validate OSBP63’s efficacy as a STING agonist, we further measured its impact on IFN-β secretion into the cell supernatant using the Quantikine^®^ Human IFN-β Immunoassay Kit, given the critical role of IFN-β as an anti-tumor effector following STING activation. The results demonstrated that OSBP63 markedly enhanced IFN-β secretion in the cells (Figure 4b), confirming its activation of STING, with a stronger effect compared to diABZI.

The significant advantage of OSBP63 over diABZI in chemokine induction suggests that it can not only robustly activate the STING pathway but also reshape the tumor microenvironment by strongly recruiting effector immune cells, thereby supporting the development of combination therapies with immune checkpoint inhibitors and other treatments. Consequently, these findings enhance the relevance of this study within the broader context of STING-targeted immunotherapy.

### 3.4. Validation of OSBP63 Binding to STING Proteins in Live Cells

**Figure 5:**
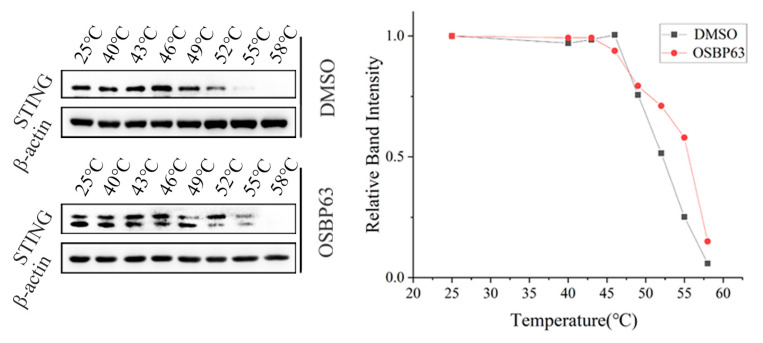
4T1 cells were treated with OSBP63 (1 µM, 24 h). DMSO was used as a vehicle control. Cells were harvested, and protein stability was assessed by the cellular thermal shift assay (CETSA). Protein band intensities were quantified with ImageJ. The melting curves of STING protein were plotted, and the correlation between the relative abundance of STING protein and temperature was further analyzed.

To confirm the binding of OSBP63 to the STING target, we employed cellular thermal shift assay (CETSA) in conjunction with protein blotting analysis. CETSA is an innovative technique for identifying drug targets. Drug binding to target proteins typically results in stabilization, reducing susceptibility to thermal denaturation [37]. We assessed the binding affinity of OSBP63 to STING by examining the thermal stability of STING proteins. Heat exposure of 4T1 cells treated with OSBP63 (1 μM) for 24 h significantly enhanced STING thermal stability within 49–55 °C compared to the DMSO control (Figure 5), indicating a strong affinity of OSBP63 for STING. This discovery not only confirms the direct interaction between OSBP63 and STING but also underscores OSBP63’s potential as a promising therapeutic agent.

### 3.5. Investigation of OSBP63 in a Transplantable Mouse Breast-Cancer Model

**Figure 6:**
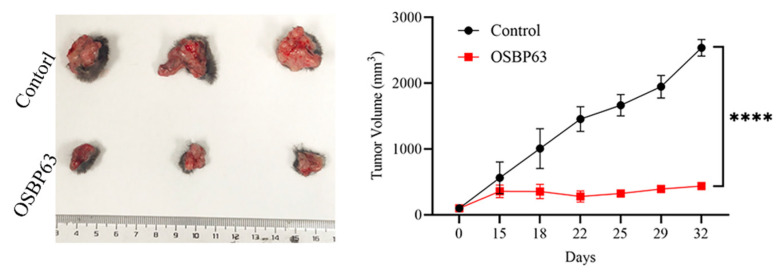
Tumor growth in mice treated with OSBP63. *C57BL/6J* mice bearing a single subcutaneous E0771 breast tumor (~100 mm^3^) received intratumoral OSBP63 (5 mg/kg). Tumor volumes were measured over time and plotted as a line graph. (*n* = 3; **** *p* < 0.0001 vs. control).

We assessed the therapeutic efficacy of OSBP63 in the E0771 mouse mammary-carcinoma model. Orthotopic tumors were generated by injecting 2 × 10^6^ E0771 cells into the mammary fat pad of female *C57BL/6J* mice. When tumors reached ~100 mm^3^, OSBP63 (5 mg/kg) was given via intratumoral injection (i.t.). As shown in Figure 6, intratumoral OSBP63 significantly inhibited tumor growth, demonstrating potent antitumor activity.

### 3.6. In Vitro Mechanistic Study of OSBP63 Combined with PTX

**Figure 7:**
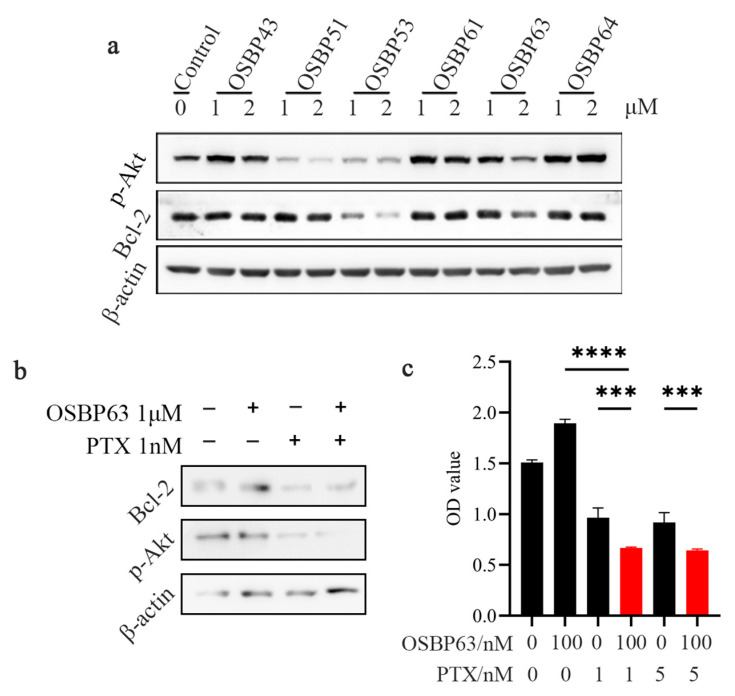
(**a**) 4T1 cells were treated with marine bromophenol (1 μM or 2 μM) for 24 h, and the expressions of p-AKT and BCL-2 were analyzed by Western blot (The band quantitative analysis graph is presented in Supplementary Figure S5). (**b**) OSBP63 (1 μM) was co-treated with PTX (1 nM) for 24 h in 4T1 cells, and the expressions of p-AKT and BCL-2 were analyzed by Western blot (The band quantitative analysis graph is presented in Supplementary Figure S5). (**c**) MTT assay of 4T1 cell viability following OSBP63 (100 nM) combined with PTX (1 nM or 5 nM). *n* = 3; *** *p* < 0.001, **** *p* < 0.0001 vs. single-agent treatment.

Accumulating evidence shows that STING agonists synergize with checkpoint inhibitors (CPIs) such as cytotoxic T-lymphocyte-associated protein 4 (CTLA4) or programmed death protein 1 (PD-1), producing stronger antitumor effects than either agent alone [38,39]. Because the AKT pathway is a central survival signal downstream of vascular growth factors, Jeong et al. reported that co-administration of a STING agonist (e.g., cGAMP) with an AKT inhibitor markedly enhanced antitumor activity [40]. Specifically, AKT inhibition was observed to induce apoptosis in tumor endothelial cells within spontaneous tumors—a response absent when either cGAMP or an AKT inhibitor was used separately. These findings underscore the complex crosstalk between STING and AKT signaling pathways. In breast cancer cell models, PTX effectively inhibits AKT signaling by reducing AKT phosphorylation [41]. Our additional data on the combination of PTX with STING agonists further support this interaction. Western blotting (Figure 7a) showed that OSBP63 alone did not alter AKT phosphorylation. In contrast, co-treatment with OSBP63 and PTX markedly suppressed AKT activity and strongly down-regulated the anti-apoptotic protein BCL-2 (Figure 7b). Consistently, an MTT assay confirmed that the OSBP63-PTX combination promoted apoptosis in cancer cells (Figure 7c).

### 3.7. In Vivo Antitumor Efficacy of OSBP63 Combined with PTX

**Figure 8:**
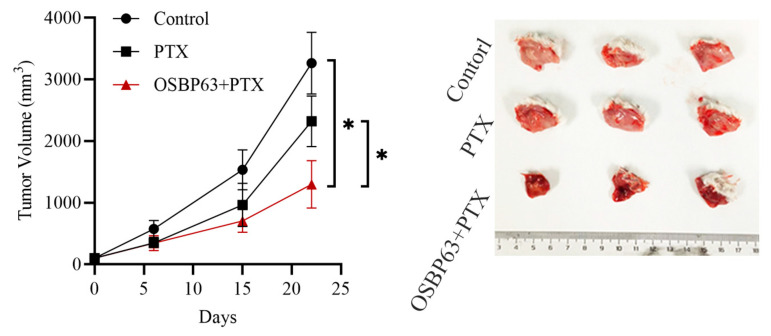
Evaluation of tumor growth in mice treated with OSBP63 in combination with PTX. BALB/c mice bearing a single subcutaneous 4T1 breast tumor (~100 mm^3^) received intravenous PTX (2 mg/kg) or a combination of OSBP63 (5 mg/kg) plus PTX (2 mg/kg). Tumor volumes were measured over time and plotted as mean values on a line graph. (*n* = 3; * *p* < 0.05 vs. control; * *p* < 0.05 vs. PTX).

Considering that STING agonists can improve the tumor immune microenvironment by driving dendritic cell maturation, enhancing cytotoxic T cell (CTL) infiltration and regulating tumor vascular remodeling [16], and the cytotoxic effect of PTX can further release tumor antigens to amplify the immune response. We assessed the antitumor effect of OSBP63 combined with PTX in an orthotopic breast cancer model. Orthotopic breast tumors were generated by injecting 5 × 10^5^ 4T1 cells into the fourth breast fat pad; treatment began 5 days later. OSBP63 (5 mg/kg) and PTX (2 mg/kg) were administered intravenously, with saline as the control. As shown in Figure 8, the OSBP63 + PTX regimen produced significantly greater tumor suppression than the control. In summary, these data indicate that the combination of OSBP63, a non-nucleotide STING agonist, and PTX operates via a dual synergistic mechanism—direct cytotoxicity (AKT inhibition and apoptosis induction) and immune regulation—and generates significant anti-tumor activity in breast cancer models. These findings support the potential to enhance therapeutic effects in clinical treatment.

## 4. Conclusions

Although STING activation is a promising strategy for cancer immunotherapy, the development of small-molecule STING agonists is hindered by poor pharmacokinetics, safety concerns, and the absence of agents that have progressed to phase III trials. Here, we identify OSBP63, a kind of bromophenol compound, as a potent STING agonist that directly binds the adaptor protein, robustly activates the cGAS–STING pathway, and elicits downstream cytokine production to suppress tumor growth. OSBP63 also synergizes with the AKT inhibitor PTX to enhance pro-apoptotic signaling and markedly inhibit tumor progression in murine models. Mechanistically, we propose that bromophenols, whose symmetrical scaffolds span approximately five to six atoms across the STING dimer interface, function as small-molecule aptamers that restore innate immune surveillance. These findings not only demonstrate the therapeutic potential of OSBP63 but also provide a structural blueprint for the rational design of next-generation STING agonists.

## Data Availability

The original contributions presented in this study are included in the article/Supplementary Materials. Further inquiries can be directed to the corresponding authors.

## Supplementary Materials

The following supporting information can be downloaded at: https://www.mdpi.com/article/10.3390/cimb48010061/s1.
