# Supplementary material for: Marine Bromophenol Derivatives as a Novel Class of Potent Small-Molecule STING Agonists
Source: Curr Issues Mol Biol. 2026 Jan 5;48(1):61. doi: 10.3390/cimb48010061 (PMC12839989; doi:10.3390/cimb48010061)
Supplement: Supplementary file 1 [file cimb-48-00061-s001.zip › cimb-4021390-supplementary.pdf]

Supplementary Information

Marine Bromophenol Derivatives as a Novel Class of Potent Small-Molecule STING Agonists

Manqing Tang [3,†], Qiuhui Guo [3,†], Ping Wang [3], Yunfei Li [3,*] and Bo Jiang [1,2,*]

[1] *Laboratory of Experimental Marine Biology, Institute of Oceanology, Chinese Academy of Sciences, Qingdao, 266000, China*

[2] *Nantong Zhong Ke Marine Science and Technology R&D Center, Nantong, China*

[3] *College of Chinese Materia Medica, Tianjin University of Traditional Chinese Medicine, Tianjin 301617, China*

† These authors contributed equally to this work.

[*] Corresponding author: liyunfei@tjutcm.edu.cn; jiangbo@qdio.ac.cn

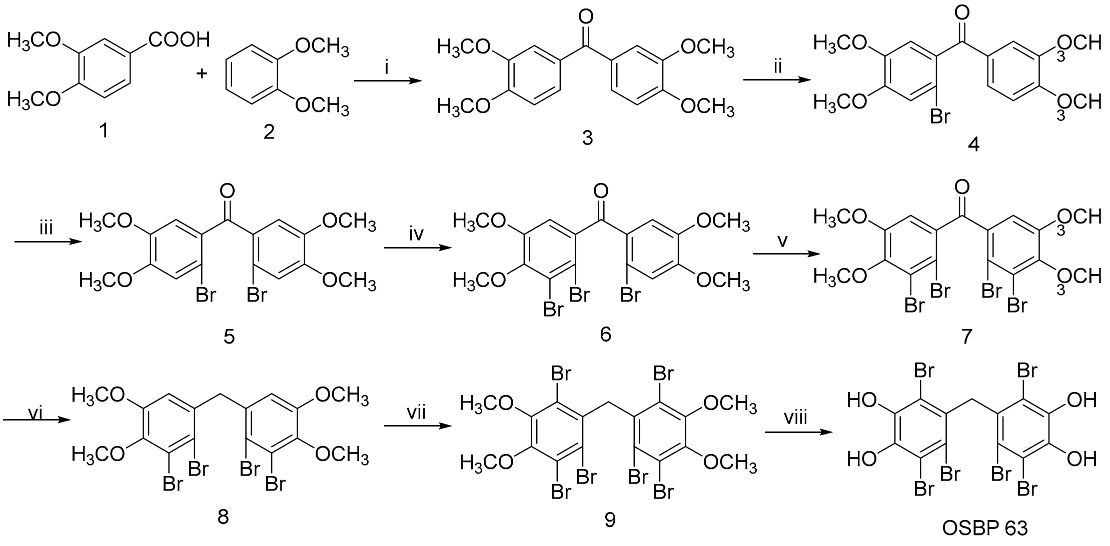

Figure S1. Reagents and conditions: (i) PPA, 80 oC, 1 h; (ii) Br2 (1.1 eqv.), CH2Cl2, r.t.; (iii) Br2 (2 eqv.), AcOH, r.t.; (iv) Br2 (1.1 eqv.), AlCl3, AcOH, 40 oC; (v) Br2 (3 eqv.), AlCl3, AcOH, 80 oC; (vi) Et3SiH, CF3COOH, r.t.; (vii) NBS (2 eqv.), con. H2SO4, 0 oC- r.t.; (viii) BBr3, dry CH2Cl2, r.t., 4h.

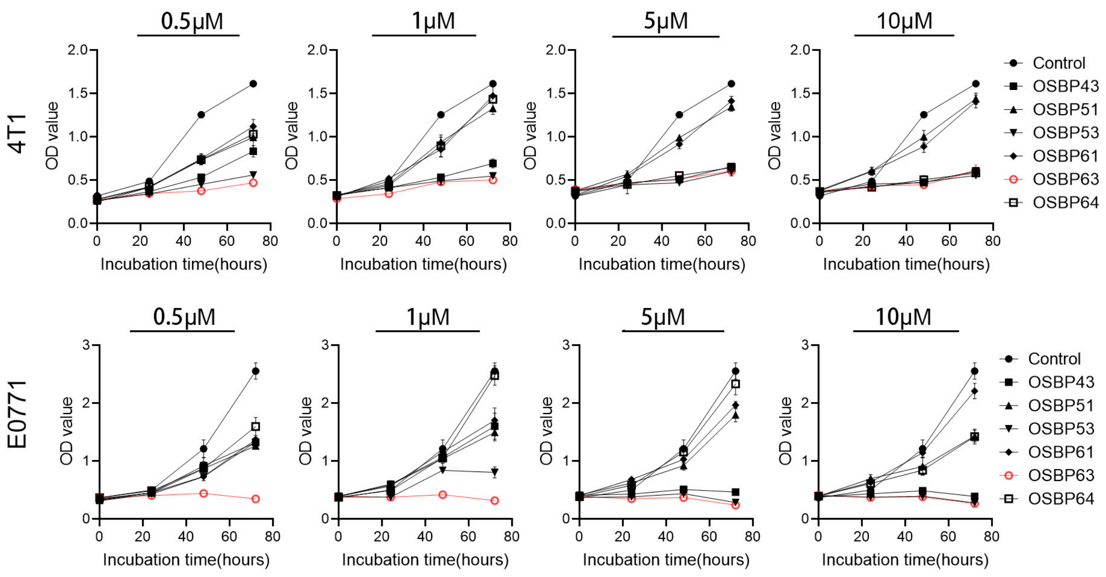

Figure S2. Marine bromophenol derivatives were exposed to 4T1 and E0771 cell lines at different concentrations (0.5, 1, 5 and 10 μM) to study the antiproliferative activity of Marine bromophenol derivatives on breast cancer cells. Cell viability was determined by the MTT assay after incubation for 0, 24, 48 and 72 hours. (n = 3)

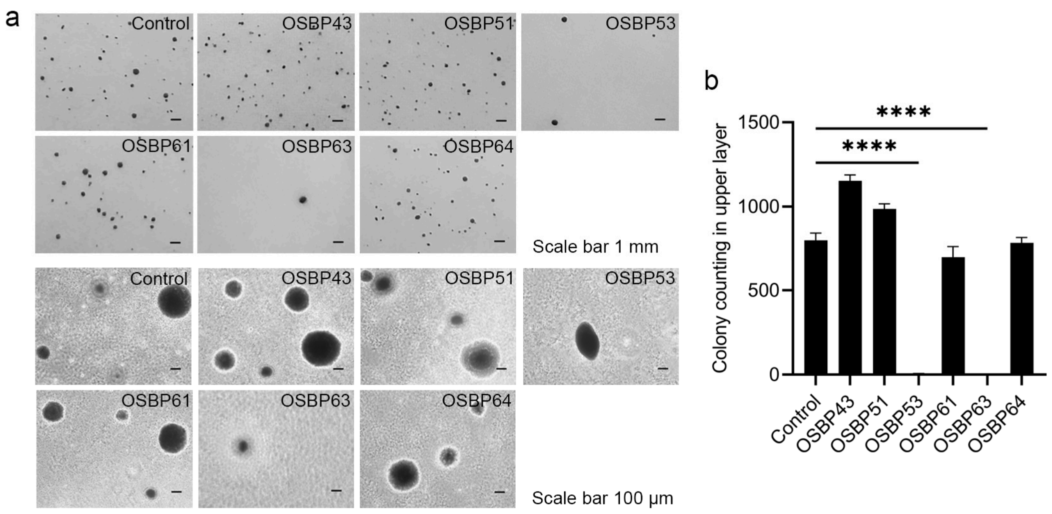

Figure S3. Effects of marine bromophenol derivatives on colony formation of 4T1 cells. a. Scanning and microscopic images of colony formation following treatment of 4T1 cells with 2μM of marine bromophenol derivatives; b. Statistical graph of the number of 4T1 cell colonies. (n=3, **** P<0.0001 compared to Control.)

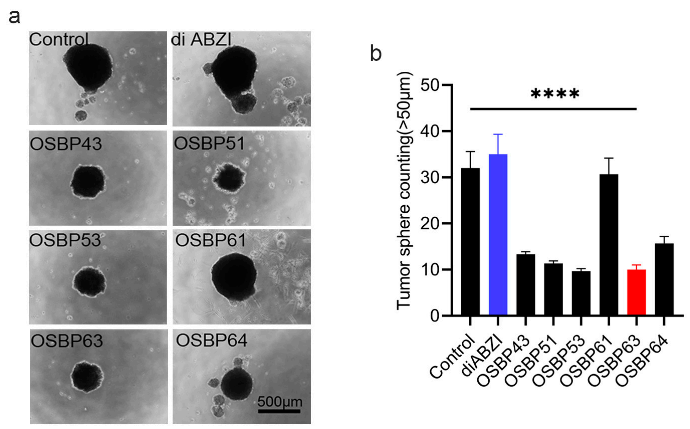

Figure S4. Effects of marine bromophenol derivatives on 4T1 cell spheroid formation capacity. a. Scanning images of 4T1 cells treated with 2 μM marine bromophenol derivatives; b. Statistical graph of 4T1 cell spheroid counts. (n=3, **** P<0.0001 compared to Control.)

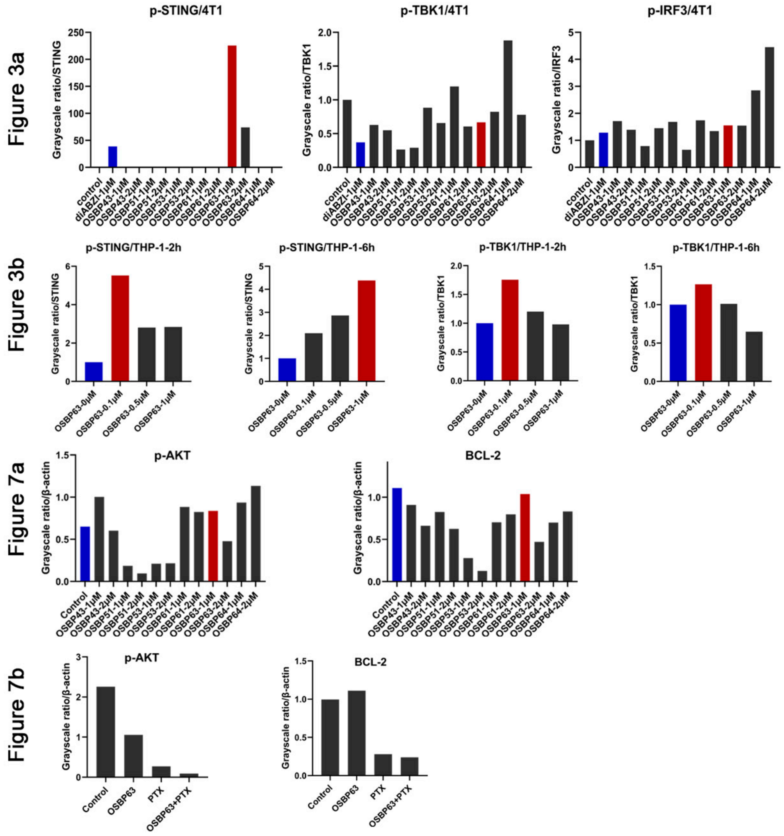

Figure S5. All quantitative data graphs of the WB bands. Quantitative data graphs for all WB bands. The markings in the figure correspond exactly to the figure numbers in the text.
